# Neurorétinite unilatérale: une manifestation rare du syndrome post streptococcique

**DOI:** 10.11604/pamj.2014.19.120.5294

**Published:** 2014-10-01

**Authors:** Said Iferkhass, Adil Elkhoyaali, Fouad Elasri, Karim Reda, Abdelkader Laktaoui, Abdelbar Oubaaz

**Affiliations:** 1 Service d'Ophtalmologie, Hôpital Militaire MY Ismail, Meknes, Maroc; 2 Service d'Ophtalmologie, Hôpital Militaire d'Instruction Mohamed v, Rabat, Maroc

**Keywords:** Vascularite, œdème papillaire, hémorragies rétiniennes, neurorétinite, érythème noueux, streptocoque, Vascularitis, papilledema, retinal haemorrhages, neuroretinitis, erythema nodosum, streptococcus

## Abstract

La streptocoque β hémolytique est responsable de plusieurs complications d'origine immunitaire, de localisation cardiaque, articulaire, dermatologique, rénale, cérébrale et oculaire. Nous rapportons le premier cas au Maroc d'une uvéite postérieure unilatérale post- streptococcique présentant une vascularite rétinienne avec œdème papillaire et des hémorragies rétiniennes en tache profondes et superficielles. Notre patient âgé de 56 ans, ayant comme antécédents pathologiques une notion d'angine et d’ érythème noueux à répétition d'origine streptococcique. Le bilan étiologique d'uvéite est revenu normal. En revanche, le titrage des anticorps antistreptococciques était significativement élevé: 430 IU /ml. Le patient a été mis sous amoxicilline protégée à une dose de 2 g par jour pendant 10 jours, associée à une corticothérapie à dose régressive sur un mois. L’évolution était favorable avec récupération totale de l'acuité visuelle (AV) et disparition des lésions neurorétiniennes

## Introduction

Le syndrome post-streptococcique est une maladie auto-immune systémique [1, 2]. Les Manifestations peuvent inclure la fièvre rhumatismale, l'arthrite réactionnelle, la glomérulonéphrite, et l′érythème noueux [1]. En raison de la rareté des cas, l′uvéite post-streptococcique n′a pas été bien reconnue par les ophtalmologues. Actuellement des cas d'uvéite antérieure non granulomateuse et, moins fréquemment, d'uvéite postérieure avaient été signalées dans la littérature [1, 3, 4]. Des cas de vascularite rétinienne et papillophlebite, même s'ils sont très rares, ont été également rapportés [1, 2, 5]. Ici, nous décrivons le premier cas d′uvéite postérieure post-streptococcique au Maroc, qui a présenté une neurorétinite unilaterale sans inflammation antérieure ou vitréenne associée.

## Patient et observation

**Figure 1:**
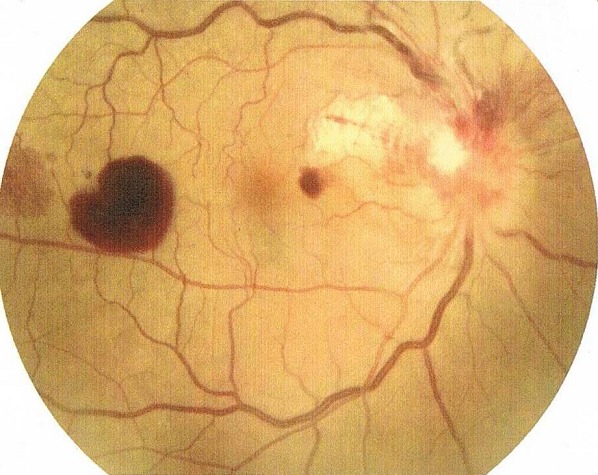
Rétinographie: flou papillaire, des taches hémorragique en flammèches épi et péri-papillaire, une lésion blanchâtre péri-papillaire temporale bien limitée et des hémorragies rétiniennes en taches profondes et superficielles

**Figure 2:**
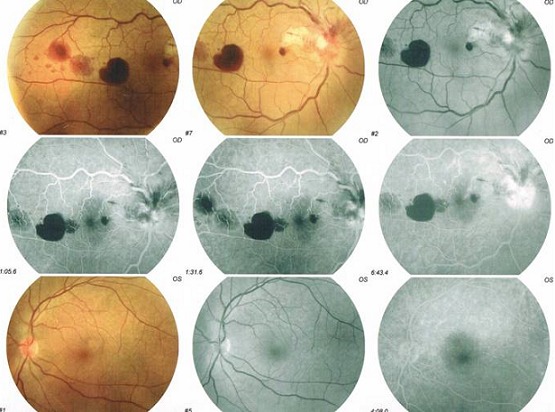
Angiographie à la fluorescéine. OD: œdème papillaire, vascularite, taches hémorragique en flammèches épi et péri-papillaire et hémorragies rétiniennes en tache profondes et superficielles. OG: aspect normal.

Patient âgé de 56 ans, ayant comme antécédents pathologiques une notion d'angine et d’érythème noueux à répétition durant trois ans auparavant. Hospitalisé à deux reprises dans le service de dermatologie où l'identification de streptocoque ß hémolytique a été faite sur des prélèvements au niveau de la gorge. Il a été mis sous traitement à base d'injection intra musculaire de Pénicilline G retard bimenstruelle. Trois mois après l'arrêt de traitement, le patient a présenté une baisse d'acuité visuelle de l’œil droit (OD), sans douleur ni rougeur oculaire, associée à des arthralgies au niveau des deux poignets. L'acuité visuelle est chiffrée à 2/10 P6. Le segment antérieur et le tonus oculaire sont strictement normaux. L'examen du fond d’œil (Figure 1) révèle une neurorétinite avec flou papillaire, des taches hémorragiques en flammèches épi et péripapillaires, une lésion blanchâtre péripapillaire temporale bien limitée et des hémorragies rétiniennes en tache profondes et superficielles, sans réaction inflammatoire du vitré associée. L'angiographie à la fluorescéine (Figure 2) confirme l’œdème papillaire et montre la présence d'une vascularite rétinienne. Le champ visuel effectué malgré une acuité visuelle basse montre un scotome profond diffus. Le PEV objective un léger allongement du temps de latence en P100 à 128 ms. L'examen clinique de l’œil adelphe est normal. Un bilan étiologique exhaustif a été demandé. Le scanner cérébral et orbitaire est sans anomalie visible. La vitesse de sédimentation est normale à 25 mm/h, mais la protéine C- réactive (CRP) est significativement élevée à 9,1mg/l. Le dosage de l'enzyme de conversion de l'angiotensine, la glycémie à jeun et le bilan phosphocalcique est strictement normal. La radiologie pulmonaire est sans particularité et la recherche de BK dans les crachats est revenue négative.

**Figure 3:**
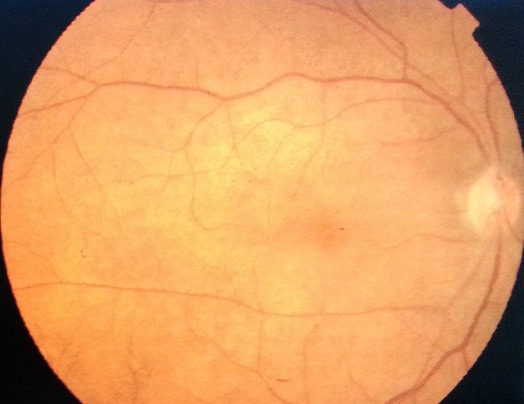
Rétinographie après traitement de l'OD: disparition des lésions neurorétiniennes

Les sérologies: VIH, cytomégalovirus, herpétique, toxoplasmique, Lyme et syphilitique, étaient négatives. Le bilan immunitaire (anti nucléaire, anti SSA, anti SSB, anti DNA natif, antiSm, RNP, ANCA et facteur rhumatoïde) était normal. Le titrage d'antistreptolysine O (ASLO) était significativement élevé à 430 UI/mL. Le diagnostic d'une neurorétinite post streptococcique fut retenu. Le patient a été mis sous amoxicilline protégée à une dose de 2 g par jour pendant 10 jours, associée à une corticothérapie à dose régressive sur un mois. L’évolution était favorable avec récupération de l'AV à 10/10 et disparition des lestions neurorétiniennes (Figure 3). Le patient a été suivi en consultation pendant quatre ans et aucune récidive n'a été constatée.

## Discussion

Le syndrome post-streptococcique (PSS) inclut toute inflammation systémique non suppurative survenant après une infection streptococcique [6]. Les manifestations courantes de ce syndrome sont d'origine immunitaire et comprennent la fièvre rhumatismale aiguë, la glomérulonéphrite, l’érythème noueux, et l′arthrite réactive. Les uvéites post-streptococciques (PSU), qui font partie actuellement du syndrome post streptococcique [6, 7], sont très rare et souvent bilatérales [1, 3, 4, 8]. Elles peuvent être la seule manifestation de PSS [3, 9] ou être associées aux autres manifestations post streptococciques, comme c'est le cas de notre patient. Au cours des 20 dernières années, et au mieux de nos connaissances, une soixantaine de cas ont été rapportés dans la littérature mondiale [1–6, 8, 9], sous forme de cas cliniques ou séries très courtes, auxquelles nous ajoutons notre cas qui présente une neurorétinite unilatérale. L'uvéite post-streptocoque inclut typiquement une uvéite antérieure non granulomateuse [1, 4, 8] qui est associée dans plus d'un tiers des cas à une atteinte du pôle postérieur [1, 3, 8]. L'atteinte postérieure est rare et implique des rétinites [1], des vascularites rétiniennes [2, 3, 7], des lésions choroïdiennes [1, 3, 5], et rarement des atteintes papillaires [1–5]. Le tableau clinique chez notre patient, est une neurorétinite de l’œil droit avec comme particularité, la présence de multiples hémorragies rétiniennes en taches, superficielles et profondes, sans réaction inflammatoire vitréenne associée. Cette présentation clinique plus alarmante associée à une histoire d'angine et d’érythème noueux post streptococcique, est rarement rapportée. En effet, seulement trois cas cliniques rapportés sont similaire à notre cas [2, 4, 5].

Bien que Le rôle des ASLO dans le diagnostic des uvéites post streptococciques reste controversé [8], les publications précédentes sur PSU [1, 2, 4, 8, 9] ont fondé le diagnostique sur le titrage des ASLO seul. La grande variabilité de titrage des ASLO interdit le développement d'un indice de sensibilité et spécificité pour les ASLO comme test diagnostic définitif aux uvéites post-streptococciques. Cependant, sa présence élevée ajoute, toutefois, une pertinence forte au diagnostic [8]. Il apparait nécessaire avant de retenir le diagnostic de PSU de mener une enquête étiologique afin d’éliminer les autres affections donnant la même présentation clinique, en particulier la tuberculose, la syphilis, la toxoplasmose, la sarcoïdose, la maladie de Behcet, l'herpès virus, la cytomégalovirus, Epstein-Barr virus, maladie de Lyme et les autres maladies auto immunes. Chez notre patient, le diagnostic repose sur l'association des critères suivants: les antécédents d'une infection streptococcique (angine et d’érythème noueux à répétition durant trois ans auparavant,et l'isolement de streptocoque pyogène sur les prélèvement de la gorge), le titrage d'ASLO élevé, et la normalité du bilan à la recherche d'autres causes d'uvéite.

Bien que la physiopathologie de l′uvéite post-streptococcique n′a pas été complètement élucidé, la plupart des auteurs postulent pour la présence d'une réaction croisée, entre les antigènes microbiens et les antigènes du soi, structurellement proches et générant des anticorps circulants chez des patients génétiquement prédisposés [5, 8] D'après Adamus G. et al [10] une forte prévalence de divers auto- anticorps chez les patients atteints de neuro-rétinopathie suggère une activation polyclonale du système immunitaire humoral. Les principaux auto-anticorps identifiés, sont diriges contre des enzymes de la glycolyse classiques impliqués dans la production d′énergie, comme l’énolase, le glycéraldéhyde 3-phosphate déshydrogénase (GAPDH) [10]. Ce sont des protéines multifonctionnelles et fortement antigéniques, qui sont toutes exprimées de manière intracellulaire, mais également sur la surface des cellules neuronales. Ces protéines ainsi que, l'aldose sont également exprimés sur les streptocoques pyogènes et leur similitude avec les enzymes de la glycolyse neuronales humaines, pourrait expliquer une réponse immunitaire à réaction croisée contre ces protéines [10].

Le traitement des uvéites post-streptococciques est un sujet de controverse [3, 8]. La plupart des auteurs recommandent en cas d'atteinte antérieure simple un traitement par corticoïde topique alors que la corticothérapie par voie orale est réservée pour les formes avec participation postérieure [1, 4, 8]. Toutefois, des preuves anecdotiques suggèrent que l′amygdalectomie peut réduire la fréquence et la gravité des PSU récurrentes [7, 8], en supprimant la plupart des réservoirs. Certains auteurs recommandent que l′infection streptococcique active ou résiduelle doit être régulièrement traitée par la pénicilline orale [7] alors que d'autres préconisent la pénicilline seulement aux patients qui n′ont pas répondu au traitement initiale de l′uvéite [3]. En l'absence d'un protocole codifié nous avons introduit avec la corticothérapie orale une antibiothérapie à base d'amoxicilline protégée. A l'exception de quelques cas récidivants, le pronostic après traitement est souvent bon, comme le montre notre cas.

## Conclusion

L'uvéite post streptococcique y compris la neurorétinite est rare. Elle doit être évoquée devant une histoire d'angine ou la présence d'un autre syndrome post streptococcique associé. Le pronostic est généralement bon après traitement.

## References

[1] Ur Rehman S, Anand S, Reddy A, Backhouse OC, Mohamed M, Mahomed I (2006). Poststreptococcal syndrome uveitis: a descriptive case series and literature review. Ophthalmology..

[2] Han J, Lee SC, Song WK (2012). Recurrent Bilateral Retinal Vasculitis as a Manifestation of Post-streptococcal Uveitis Syndrome. Korean J Ophthalmol..

[3] Tinley C, Van Zyl L, Grötte R (2012). Poststreptococcal syndrome uveitis in South African children. Br J Ophthalmol..

[4] Viel A, Kolyvras N, Catherine J, Relvas L, Judice M, Caspers L, Willermain F (2011). Post-streptococcal uveitis. Journal français d’ophtalmologie..

[5] De Smet MD (2009). Papillophlebitis and uveitis as a manifestation of post-streptococcal uveitis syndrome. Eye.

[6] Kais Abderrahim, Ahmed Chebil, Yosra Falfoul, Mejda Bouladi, Leila El Matri (2012). Granulomatous uveitis and reactive arthritis as manifestations of post-streptococcal syndrome. International ophthalmology.

[7] Leiba H, Barash J, Pollack A (1998). Poststreptococcal uveitis. Am J Ophthalmol.

[8] Gallagher MJ, Muqit MM, Jones D, Gavin M (2006). Post-streptococcal uveitis. Acta Ophthalmol Scand.

[9] Fretzayas M, Moustaki E, Stefos E, Dermitzaki P, Nicolaidou (2010). Uveitis: an isolated complication of post-streptococcal syndrome. Annals of Tropical Peadriatrics.

[10] Grazyna Adamus, Lori Brown, Jade Schiffman, Alessandro Iannaccone (2011). Diversity in autoimmunity against retinal, neuronal,and axonal antigens in acquired neuro-retinopathy. J Ophthal Inflamm Infect.

